# Thiol-Reactive or Redox-Active: Revising a Repurposing Screen Led to a New Invalidation Pipeline and Identified a True Noncovalent Inhibitor Against Papain-like Protease from SARS-CoV-2

**DOI:** 10.1021/acsptsci.4c00325

**Published:** 2024-10-04

**Authors:** Maria Kuzikov, Stefano Morasso, Jeanette Reinshagen, Markus Wolf, Vittoria Monaco, Flora Cozzolino, Simona Golič Grdadolnik, Primož Šket, Janez Plavec, Daniela Iaconis, Vincenzo Summa, Angela Corona, Annalaura Paulis, Francesca Esposito, Enzo Tramontano, Maria Monti, Andrea R. Beccari, Candida Manelfi, Björn Windshügel, Philip Gribbon, Paola Storici, Andrea Zaliani

**Affiliations:** † Fraunhofer Institute for Translational Medicine and Pharmacology ITMP, Discovery Research ScreeningPort, Schnackenburgallee 114, 22525 Hamburg, Germany; ‡ School of Science, Constructor University, Campus Ring 1, 28759 Bremen, Germany; § Protein Targets for Drug Discovery Lab, Elettra-Sincrotrone Trieste S.C.p.A., SS 14 - km 163,5 in AREA Science Park, 34149 Basovizza, Trieste, Italy; ∥ Department of Chemical and Pharmaceutical Sciences, University of Trieste, Via Licio Giorgeri 1, 34127 Trieste, Italy; ⊥ Department of Chemical Sciences, University of Naples “Federico II’, Comunale Cinthia 26, 80126 Naples, Italy; ▲ CEINGE Advanced-Biotechnologies “Franco Salvatore”, Via Gaetano Salvatore 486, 80145 Naples, Italy; ∇ Laboratory for Molecular Structural Dynamics, National Institute of Chemistry, Hajdrihova 19, 1000 Ljubljana, Slovenia; ○ Slovenian NMR Center, National Institute of Chemistry, Hajdrihova 19, 1000 Ljubljana, Slovenia; ◆ EXSCALATE - Dompé Farmaceutici SpA, via Tommaso De Amicis 95, 80131 Naples, Italy; ¶ Department of Pharmacy, University of Naples “Federico II”, Via D. Montesano, 49 80131 Naples, Italy; △ Dipartimento di Scienze della vita e dell’ambiente, Cittadella Universitaria di Monserrato, SS-554, Monserrato, 09042 Cagliari, Italy

**Keywords:** SARS-CoV-2, drug repurposing, papain-like protease, redox, STD-NMR, CPI-169, GRL-0617

## Abstract

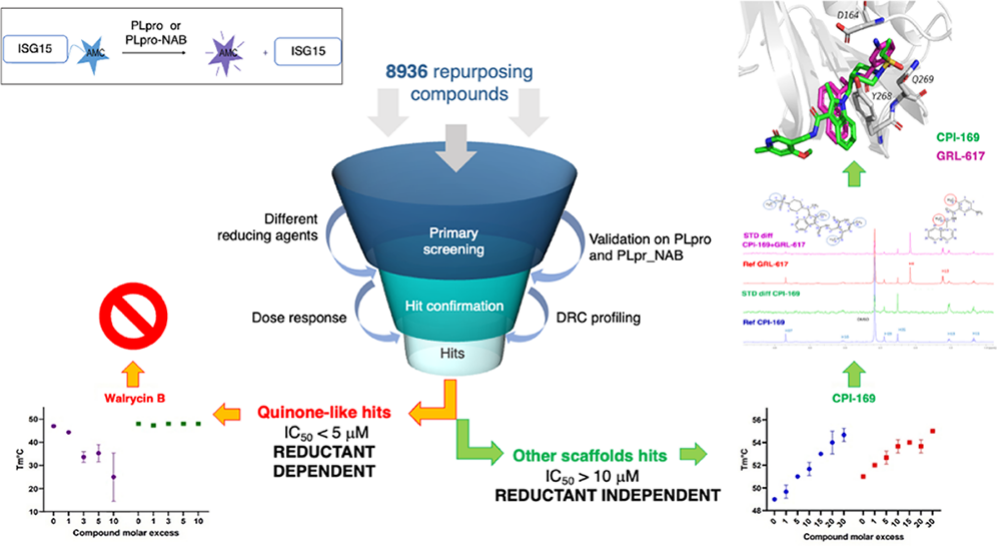

The SARS-CoV-2 papain-like protease PLpro has multiple roles in the viral replication cycle, related to both its polypeptide cleavage function and its ability to antagonize the host immune response. Targeting the PLpro function is recognized as a promising mechanism to modulate viral replication, while supporting host immune responses. However, the development of PLpro-specific inhibitors remains challenging. Comprehensive investigations utilizing enzymatic, binding studies, and cellular assays revealed the previously reported inhibitors to act in an unspecific manner. At present, GRL-0617 and its derivatives remain the best-validated compounds with demonstrated antiviral activity in cells and in mouse models. In this study, we refer to the pitfalls of the redox sensitivity of PLpro. Using a screening-based approach to identify inhibitors of PLpro’s proteolytic activity, we made extensive efforts to validate active compounds over a range of conditions and readouts, emphasizing the need for comprehensive orthogonal data when profiling putative PLpro inhibitors. The remaining active compound, CPI-169, was shown to be a noncovalent inhibitor capable of competing with GRL-0617 in NMR-based experiments, suggesting that it occupied a similar binding site and inhibited viral replication in Vero-E6 cells, opening new design opportunities for further development as antiviral agents.

After more than four years, there is still a significant effect of the SARS-CoV-2 pandemic on health and economic systems worldwide. The development of multiple vaccines against SARS-CoV-2 has improved outcomes for infected individuals.^1,2^ In addition, small molecule drugs have been introduced into the clinic, including the repurposed compound remdesivir, which inhibits the RNA-dependent RNA polymerase (RdRp), and the main protease (Mpro) inhibitor nirmatrelvir (PF-07321332), approved for application in combination with ritonavir.^3,4^ As key enzymes in SARS-CoV-2 replication, both RdRp and Mpro were initially prioritized as the first targets for the development of novel virus-specific antivirals. However, the papain-like protease (PLpro) has recently gained significant attention as the next most promising target in SARS-CoV-2 therapy, owing to its multiple roles in the maturation of viral polyproteins and antagonization of the host immune response. PLpro is responsible for the release of nsp1–4 from the viral polyprotein and, in addition, it modulates host cellular ubiquitination and ISGylation processes, both playing pivotal roles in the regulation of innate immune responses to viral infections.^5,6^

PLpro is part of nsp3, the largest nonstructural protein within the SARS-CoV-2 genome comprising multiple roles and functionally independent domains within one protein.^5^ SARS-CoV and SARS-CoV-2 PLpro share 83% sequence identity, being highly conserved within the coronavirus family^7^ and raising the possibility for the development of broad-spectrum anticoronaviridae inhibitors.^8^ Nsp3 contains five domains, named nsp3a–3e, interconnected by various linkers, followed by two transmembrane regions (TM) as well as a C-terminal Y-domain.^9^ The proteolytically active papain-like protease domain resides within the nsp3d region together with ubiquitin-like domain 2 (Ubl2). PLpro is a cysteine protease with a catalytic triad composed of Cys111–His272–Asp286, which shows recognition preferences for the LXGG↓XX motif (the arrow indicates the cleavage site).^5,10^ The substrate binding subsites S1 and S2 form a narrow tunnel responsible for recognizing two glycines, while S4 identifies the hydrophobic side chain of Leu (or Ile). S3 lacks specific residue preference due to its interaction with the peptide backbone.^11^ Additionally, PLpro has two ubiquitin-binding sites (Ub1 and Ub2) positioned distally from the active site. While SARS-CoV-2 PLpro exhibits a substrate preference for ISG15, SARS-CoV PLpro preferentially cleaves K48-linked di-Ub chains.^12,13^ Nsp3e is located at the C-terminus of PLpro and contains the nucleic acid binding (NAB) domain. SARS-CoV NAB has been demonstrated to bind ssRNA and unwind dsDNA in an ATP-independent manner, revealing a nucleic acid chaperone-like function.^5,14^

The low homology between SARS-CoV-2 PLpro and human cysteine proteases (<28% sequence identity) might suggest a limited risk of side effects associated with a selective viral protease inhibitor-based treatment. However, PLpro shares similarities with human deubiquitinating enzymes, with the closest homologues being UCH-L1, USP14, and USP7 (HAUSP).^15^ Although sequence identities are very low (11–14%), the structural topology “thumb–palm–finger” architecture is highly conserved. In particular, the catalytic triad of USP14 in the substrate-unbound state aligns well with that of the unbound state of PLpro. In contrast to USP14 and PLpro, USP7 requires substrate binding to adopt the active conformation. USP18 is the second USP of importance due to its specificity in cleaving ISG15 similar to what is observed for SARS-CoV-2 PLpro. Consequently, PLpro inhibitors may not only impact the host antiviral immune response but also interfere with the overall protein homeostatic balance in cells.

SARS-CoV-2 papain-like protease (PLpro) is a recognized drug target due to its pivotal role in viral replication. However, its unique binding pocket has posed significant challenges to inhibitor development. So far, most of the reported PLpro inhibitors include compounds that react with the active site cysteine (Cys111), zinc conjugate inhibitors, thiopurine compounds, natural products, and allosteric naphthalene inhibitors.^16,17^ Naphthalene-based ligands represent a large class of PLpro inhibitors with available structure–activity relationship (SAR) information. Notably, these encompass the well-described noncovalent inhibitor GRL-0617, which has demonstrated antiviral activity in Vero-E6 cell infection models.^17^ It occupies subsites S3 and S4 within the BL2 groove. This binding site, which competes for the ISG15 substrate, has emerged as a promising hot spot for antiviral drug discovery.^18^ Very recent advancements have yielded new GRL-0617-based derivatives, resulting in the identification of two promising candidates with potent antiviral activity in mice, validating PLpro as a viable drug target.^19,20^ Acriflavine, another promising inhibitor, was reported to have nanomolar potency against SARS-CoV-2 PLpro in enzymatic, cell-based, and in vivo studies.^21^ By NMR and MX structural analysis, two proflavines were demonstrated to occupy the substrate-binding pocket of PLpro. Natural products have also been described as SARS-CoV and SARS-CoV-2 PLpro inhibitors; these include tanshinones, geranylated flavonoids, and polyphenols.^5,22^ However, recent publications have raised doubts about the mechanism of action of many proposed inhibitors. Ma and Wang revealed limitations of reported inhibitors by profiling them through a series of enzymatic, binding, and cellular activity assays, which invalidated compounds such as the tanshinone family, YM155, SJB2-043, 6-thioguanine, and 6-mercaptopurine. The study suggests that many of the so-far reported PLpro inhibitory compounds may appear active in biochemical assays due to unspecific reactivity with PLpro, which affects its stability.^23^

In this study, we report the careful evaluation of the results of a biochemical high-throughput screen conducted with nearly 9000 compounds, from which the instability of PLpro in the presence of oxidoreduction-sensitive compounds was revealed. We delineate the reanalysis efforts that resulted in the identification of CPI-169. The data indicate that CPI-169, while less potent than the renowned GRL-0617, exhibits a similar binding site within the protein and a mild inhibition of viral growth without cytotoxicity, suggesting it as a promising scaffold for the development of a new class of molecules.

## Material and Methods

### Protein Expression and Purification

The PLpro catalytic domain construct WT (PLpro) and the inactive mutant of the catalytic Cystein111 (PLpro C111S) cloned in the *pMCSG53* vector were kindly provided by Andrzej Joachimiak (Argonne National Laboratory, Argonne). Expression was further optimized in *E. coli* BL21(DE3) by coexpressing chaperones GroEL and GroES (*pGro7* vector, TakaraBio). Cells were grown in LB medium with ampicillin (0.1 mg/mL), chloramphenicol (54 μg/mL), and L-arabinose (0.5 mg/mL) and induced for PLpro expression with 0.5 mM IPTG and 10 μM ZnCl_2_ overnight at 20 °C. PLpro WT and C111S were purified from harvested cells following the protocol reported by Osipiuk et al.^24^ Purified PLpro samples were stored in 20 mM Hepes, 150 mM NaCl, 1 μM ZnCl_2_, 10 mM DTT, and pH 7.5; flash frozen in liquid N_2_; and preserved at −80 °C.

The DNA sequence encoding for the PLpro-NAB construct (1564–2047 of nsp3) was inserted into pET24b (Novagen). The Cys111Ser mutant (PLpro-NAB C111S) was obtained by site-directed mutagenesis (primers 5′-GGACAACAACAGCTATCTGGCGA and 5′-GCCCATTTGATGCTGGTC). The plasmid was transformed into BL21(DE3), grown in LB + kanamycin (50 μg/mL), and induced by 0.25 mM IPTG at 20 °C for 20 h, adding 50 mM ZnSO_4_. WT and C111S constructs were extracted from harvested cells by homogenization and soluble fractions loaded on a 5 mL HisTrap FF Crude column (GE Healthcare Life Sciences) equilibrated in binding buffer (20 mM Tris pH 8.0, 500 mM NaCl, 10 mM imidazole, 1 mM DTT). The PLpro fraction was eluted by a 0–100% gradient of elution buffer (20 mM Tris pH 8.0, 500 mM NaCl, 300 mM imidazole, 1 mM DTT). After negative IMAC, the TEV-cleaved proteins were purified in two steps: 1) IEX on a 5 mL HiTrap Q HP (GE Healthcare Life Sciences) using buffer A: 20 mM bicine (pH 9) and 2 mM DTT, and buffer B: buffer A + 1 M NaCl; 2) SEC on Superdex 200 Hiload26/600 (GE Healthcare Life Sciences) in 20 mM Tris pH 8.0, 150 mM NaCl, and 2 mM DTT. Purified fractions were concentrated to 18–25 mg/mL, aliquoted, flash frozen, and stored at −80 °C until usage.

### Characterization of PLpro Cysteine Modifications

PLpro-NAB diluted at 7 μM in ±1 mM DTT buffer was incubated for 30 min at 25 °C in the presence or absence of PD119507 or walrycin B added at a molar ratio of 1:5. The protein samples were hydrolyzed with pepsin as reported by Bocedi et al., vacuum-dried, and resuspended in 20 μL of HCOOH 0.2% in LC-MS grade Water (Waters, Milford, MA).^25^ Each sample (8 μL) was analyzed by LC-MS/MS on an LTQ Orbitrap XL instrument (Thermo Scientific, Waltham, MA) coupled to the nanoACQUITY UPLC system (Waters). Samples were concentrated onto a C18 capillary reverse-phase precolumn and fractionated onto a C18 capillary reverse-phase analytical column (250 mm, 75 μm, 1.8 μm, M-class Waters) working at a flow rate of 300 nL/min. MS/MS analyses were performed using the data-dependent acquisition (DDA) mode; after one full MS scan (mass range from 300 to 1800 *m*/*z*), the 5 most abundant ions were selected for the MS/MS scan events. Peptide identification was analyzed with Mascot software. The relative quantification of peptides containing multioxidized cysteine residues was carried out by employing the extracted ion current approach. The percentage of modification was calculated as the ratio of the total area of all species containing the specific modification to the total area of all the species containing the catalytic cysteine.

### Primary Assay and Screening Assay

In the PLpro-NAB primary screen, compounds, positive (20 μM PR619) and negative (100% DMSO) controls, were transferred to 384-well assay microplates (Corning #3820) by acoustic dispensing (Echo, Labcyte). 5 μL of SARS-CoV-2 PLpro-NAB mix was added to compound plates. Plates were sealed and incubated for 30 min at 25 °C. After the addition of 5 μL ISG15-AMC substrate (R&D Systems #UL-553), the final concentrations were 0.15 μM substrate, 1 nM SARS-CoV-2 PLpro-NAB, 20 μM compound, and 0.2 v/v % DMSO in a total volume of 10 μL/well. During assay development, Z-LRGG-AMC was also used as the substrate. The final concentration of assay components was Z-LRGG-AMC 20 μM (or in dose response), 100 nM SARS-CoV-2 PLpro-NAB, 20 μM compound, and 0.2 v/v % DMSO in a total volume of 10 μL/well. The fluorescence signal was measured after 15 min incubation with the substrate (Ex/Em340/460; Envision, PerkinElmer). Assay buffer consists of 50 mM Tris, 150 mM NaCl, 1 mM DTT, and 0.01% Tween 20 (pH 7.5). In hit confirmation and profiling, 1 mM DTT was exchanged with 1 mM l-cysteine.

Inhibition of SARS-CoV PLpro was measured using 1 nM SARS-CoV PLpro (Biomol, #SBB-DE0024) and 0.25 μM ISG15-AMC substrate. The fluorescence signal was measured after 15 min of incubation with the substrate.

### SARS-CoV-2 Mpro Inhibition Assay

The enzymatic activity of Mpro was measured as described previously.^26^ Briefly, compounds were incubated with 60 nM Mpro for 60 min, and 15 μM of DABCYL-KTSAVLQ↓SGFRKM-EDANS substrate (Bachem #4045664) was added, followed by signal detection after 15 min incubation at Ex/Em = 340/460 nm using an EnVision microplate reader (PerkinElmer). The assay buffer contained 20 mM Tris (pH 7.3), 100 mM NaCl, and 1 mM EDTA. Zinc pyrithione at 20 μM (MedChemExpress, #HY-B0572) was used as positive control, and DMSO was used as solvent control.

### Cathepsin-L Assay

Cathepsin-L cysteine protease activity was measured using the fluorometric cathepsin-L Inhibitor Screening Kit (BPSBioscience #79591). The assay was performed according to the manufacturer protocol, adapted to 384-well format with a final volume of 10 μL. Briefly, compounds were transferred in black 384-well microplates (Corning #3820) by acoustic dispensing (Echo, Labcyte). 5 μL of cathepsin-L was added and incubated for 30 min at 25 °C. The enzymatic reaction was initiated by adding 5 μL substrate. The generated AFC signal was detected after 15 min at RT using Ex/Em = 360/460 nM (Envision, PerkinElmer). Final assay concentrations of cathepsin-L and substrate were 0.01 ng/μL and 5 μM, respectively. E-64 provided in the kit was used at a final concentration of 50 μM as a positive control for cathepsin-L inhibition (100% inhibition). DMSO was used as negative control (0% inhibition).

### USP7 and USP14 Inhibition Assay

5 μL per 100 nM of USP7/USP14 (BPS Bioscience, #80364) was added to assay plates containing the test compounds. Plates were sealed and incubated for 30 min at 25 °C, followed by adding 5 μL/0.5 μM Ubiquitin-AMC (R&D Systems #U-550-050) substrate. The fluorescence signal was measured after 30 min of incubation (Ex/Em = 340/460; EnVision, PerkinElmer). Inhibition by PR619 (Merck #662141) at 200 μM was set to 100%, and DMSO was set to 0% inhibition. The assay buffer is 50 mM Tris, 150 mM NaCl, 1 mM DTT, and 0.01% Tween 20 (pH 7.5).

### Thermal Shift Assay (TSA)

TSA was performed using PLpro-NAB and PLpro (5 and 7 μM, respectively) in white 96-well PCR plates (Bio-Rad) at a final volume of 20 μL. The assay was performed in 20 mM Tris pH 7.5, 150 mM NaCl, 1 mM DTT, or l-cysteine as the reducing agent. Compounds were added at increasing concentrations with a final DMSO concentration of 2.5% and incubated for 30 min at RT. Protein Thermal Shift dye (Thermo Fisher Scientific) was added at a final concentration of 0.7× from 1000× stock, and emission of the dye at 560–580 nm was detected using a real-time PCR (CFX96, Bio-Rad) every 30 s, with a temperature gradient of 2 °C/min. Each analysis was executed in comparison to a negative control: buffer or compounds with dye, and a positive control: protein or protein with 2.5% DMSO plus the fluorophore.

### NMR

All NMR experiments were recorded on a Bruker Avance Neo 600 MHz spectrometer equipped with a cryoprobe at the Slovenian NMR Centre of the National Institute of Chemistry, Ljubljana, Slovenia. Spectra were recorded at 298 K by using the pulse sequences included in the Bruker TopSpin library of pulse programs. PLpro-NAB buffer was exchanged with 20 mM phosphate buffer (pH 8) and 50 mM NaCl with 10% deuterated water; compounds were dissolved in DMSO-d_6_. CPI-169 was tested at 0.5 mM in 6% DMSO-d_6_ and GRL-0617 at 0.3 mM in 6% DMSO-d_6_. The assignment of ^1^H and ^13^C chemical shifts for CPI-169 and GRL-0617 was performed using 2D experiments, including ^1^H–^1^H TOCSY with a mixing time of 0.02 s, ^1^H–^13^C HSQC, and ^1^H–^1^H tr-NOESY experiments. 1D ^1^H STD experiments^27^ were performed with different concentrations while the protein:compound ratio remained 1:100.

The STD ligand epitope mapping^28^ of CPI-169 was performed with 65 536 data points, 3520 scans, and a relaxation delay of 3 s using a ligand concentration of 0.5 mM. A selective on-resonance saturation of PLpro-NAB was used for 1 s at −0.772 ppm, with a transmitter offset referenced to 4.699 ppm. The off-resonance irradiation was applied at 30 ppm for the reference spectrum.

The STD effect was obtained by calculating the STD amplification factors (*A*_STD_):
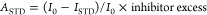
1

The ligand-binding epitope was represented with relative *A*_STD_ values normalized to the highest *A*_STD_ value (100%).

The competitive STD experiment was performed by using 0.15 mM CPI-169. Selective protein saturation was prolonged to 2 s to achieve a higher signal-to-noise ratio of STD signals at lower concentrations. First, the 1D ^1^H STD spectrum was recorded at a PLpro-NAB:CPI-169 ratio of 100:1, followed by the addition of GRL-0167 at a GRL-0617:CPI-169 ratio of 2:1 (DMSO-d_6_ 5.5%), and the second 1D ^1^H STD spectrum was assessed and the *A*_STD_ of the methyl protons was calculated and compared.

The errors in the STD amplification factor were estimated according to the formula:^29^
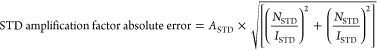


NSTD and NREF are the noise levels in STD and reference spectra. ISTD and IREF are the signal intensities in STD and reference spectra.

The tr-NOESY spectra were acquired with a spectral width of 5882 Hz, 4096 data points in t_2_, 64 scans, 128–182 complex points in t_1_, a mixing time of 250 ms, and a relaxation delay of 1.5 s.^30^ Spectra were zero-filled twice and apodized with a squared sine-bell function shifted by π/2 in both dimensions.

### Docking of CPI-169

Docking of CPI-169 was performed using GOLD version 2022.3.0 (Cambridge Crystallographic Data Centre).^31^ The PDB:7JRN X-ray structure has been used for cavity identification, from which coordinates of cocrystallized GRL-0617 and water atoms were deleted. A 6 Angs sphere has been used around GRL-0617. A 3D structure of CPI-169 was generated within MOE (Chemical Computing Group) and docked into PLpro. No specific constraints were used during docking in GOLD, and the finest generation of candidates has been used. Ten poses for each compound have been sampled, and the best CHEMPLP scores were used to identify reported poses.

### SARS-CoV-2 Viral Replication Assay in Vero-E6 GFP

Vero-E6 GFP cells were maintained in DMEM (Gibco) supplemented with 10% v/v FBS (Gibco), 0.075% Na bicarbonate (7.5% solution, Gibco), and 1× Pen-Strep (Euroclone).

The SARS-CoV-2 BetaCoV/Belgium/GHB-03021/2020 strain was kindly provided by KU Leuven and amplified in Vero-E6 GFP. All SARS-CoV-2-related work was carried out in a certified, high-containment biosafety level 3 facility at the University of Cagliari.

Vero-E6 GFP cells were seeded at 10^4^ cells/well in 96-well plates. The following day, cells were incubated with or without compounds in the presence of 2 μM P-gp inhibitor CP-100356 at different concentrations and infected with a multiplicity of infection (MOI) of 0.01. Compound GC376 was used as a positive control in the presence of the 2 μM P-gp inhibitor CP-100356. 24 h post infection, 140 mL of supernatant was collected and viral RNA was isolated using the QIAamp Viral RNA Mini Kit (QIAGEN) according to manufacturer’s instructions. Isolated RNA was then reverse transcribed, and the SARS-CoV-2 spike protein (S) gene was amplified using a Luna universal one-step quantitative real-time PCR (RT-qPCR) kit (New England BioLabs); S protein RNA levels are expressed in viral copy number.

### Data Analysis

Data analysis was performed using GraphPad Prism 8 (GraphPad Software) and ActivityBase (IDBS). Test compound results were normalized relative to respective controls, and control well outliers were eliminated according to the three-sigma method. Dose–response curves were fitted to 4-parameter logistic functions.

## Results

### Assay Development and Enzymatic Stability of SARS-CoV-2 PLpro

Being a multidomain protein, SARS-CoV-2 nsp3 comprises various functions. Usually, the individual domains of nsp3 are analyzed separately to dissect their cellular and viral function. In the case of PLpro, the nsp3d region is typically used to screen for compounds inhibiting its proteolytic activity.^32,33^ To increase the chance of finding new inhibitors, we employed an extended construct that includes not only the nsp3d region but also the NAB/nsp3e domain. This extended construct putatively allows additional interaction points with PLpro substrates as well as with inhibitors.

**Figure 1:**
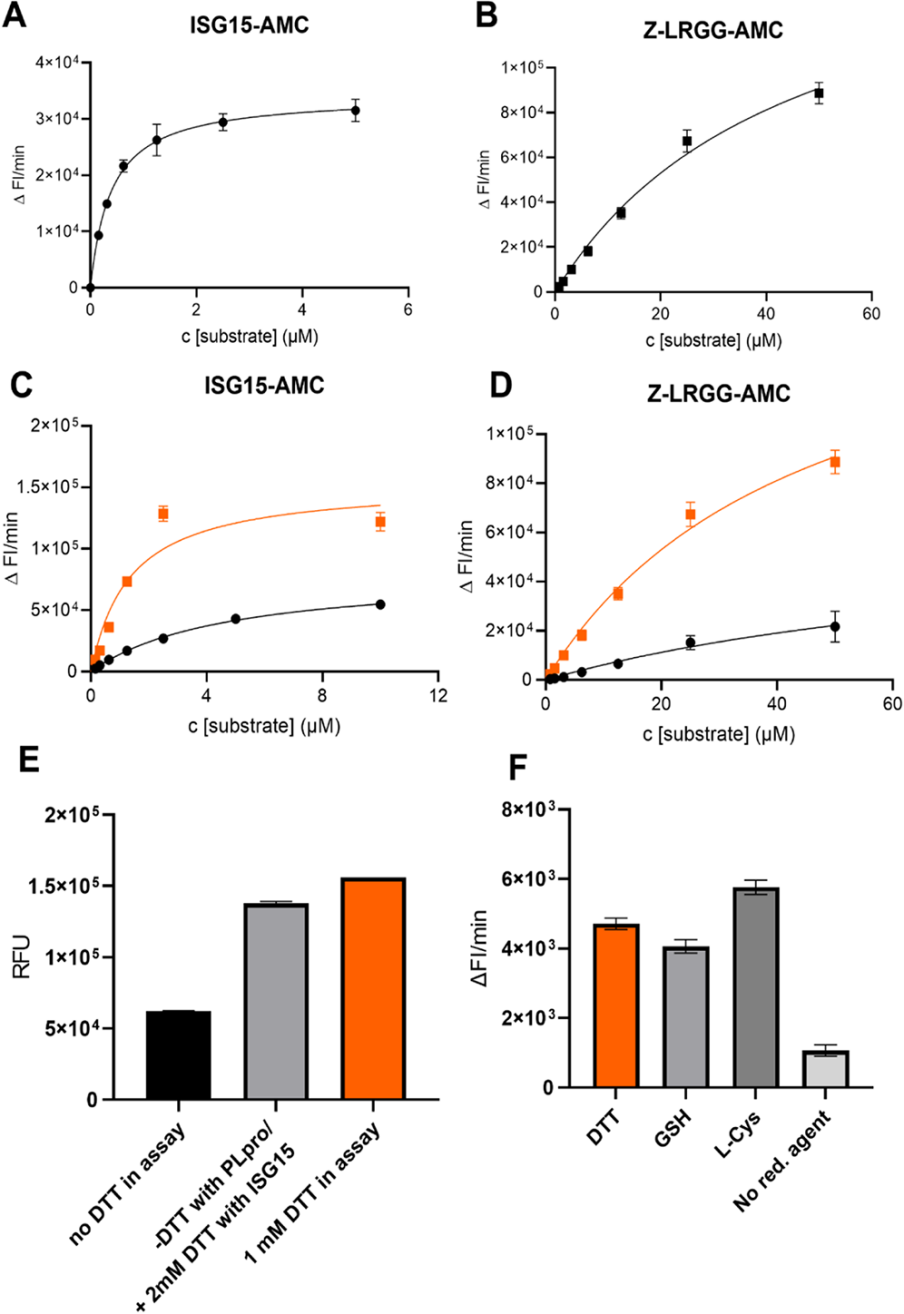
Key kinetic parameters of SARS-CoV-2 PLpro-NAB using different substrates. (A) 1 nM PLpro-NAB incubated with the biologically native substrate ISG15-AMC. (B) 100 nM PLpro-NAB incubated with the reference substrate Z-LRGG-AMC. ISG15-AMC: Vmax = 34 186/min, Km = 0.39 μM, Z-LRGG-AMC: Vmax = 173 041 /min, Km = 45.2 μM (*n* = 3, data points represent means ± SD). (C,D) Determination of PLpro-NAB activity after 30 min incubation using ISG15-AMC (C) and Z-LRGG-AMC (D) in the presence of 1 mM DTT (orange line) and absence of DTT (black line). (E) Rescue effect of DTT measured at different steps of the assay components addition. (F) Potential for the reducing agent exchange measured with reaction velocity in different assay buffers at 1 mM reducing agent concentration (*n* = 3, bars represent means ± SD).

To develop a more biologically relevant screening setup for SARS-CoV-2 PLpro_NAB, we first evaluated two reported substrates: the native-like substrate ISG15(-AMC) and the ubiquitin-derived short peptide Z-LRGG(-AMC), frequently employed in PLpro biochemical screens. The Km values obtained with the two substrates (Figure 1A,Figure 1B) align well with previously reported values.^32^ Notably, we observed no difference in Km values for ISG15-AMC between the commonly used catalytic domain construct (PLpro) and the newly generated PLpro-NAB construct (Figure S1A).

To ensure protein activity over long incubation periods, we evaluated enzyme activity after a preincubation time of 30 min before substrate addition. We observed an increase in the Km value for ISG15-AMC from 0.39 to 5 μM after 30 min preincubation. As redox sensitivity is common in cysteine proteases, we tested the impact of reducing agents, such as DTT on protein stability. The substrate turnover stabilized, with a 5-fold reduction in Km and a 2-fold increase in Vmax when ISG15-AMC was used in the presence of 1 mM DTT compared to DTT-free conditions (Figure 1C). Similar DTT effects occurred when the Z-LRGG-AMC substrate was used (Figure 1D). Moreover, the loss of activity was rescued upon the addition of 2 mM DTT simultaneously with the substrate so that the loss of activity effect that occurred during the preincubation time could be rescued with a final concentration of 1 mM DTT (Figure 1E). Knowing that the impact of a reducing agent depends on its reducing potential and that it can affect not only the protein but also its ligand/compound, we evaluated different assay buffers to ensure PLpro stability under diverse conditions. The usage of different reducing agents at 1 mM concentration showed comparable results (Figures 1F and S1B).

To better characterize the loss of enzymatic activity in the absence of DTT, we performed a mass mapping analysis. Modification in the oxidation state of the protein, particularly occurring at the catalytic cysteine Cys111, can explain the observed loss of enzymatic activity. PLpro-NAB was first incubated in the presence and absence of 1 mM DTT for 30 min at RT, and each sample was hydrolyzed with pepsin and analyzed by LC-MS/MS. A minor, but measurable, increase in dioxidated forms was observed in the DTT-free buffer, confirming our observation that DTT rescues the enzymatic activity of PLpro when added after 30 min of incubation at RT, which is only possible for the reversible mono-oxidation forms of cysteine (Table 1). Thus, we assume a high sensitivity of PLpro toward changes in redox conditions.

**Table 1:** Percentage of the Catalytic Cysteine Modification in the Presence and in the Absence of DTT Using PLpro-NAB

Oxidation state of Cys111	DTT	
	+	–
no modification	99%	98%
dioxidation	1%	2%
trioxidation	0%	0%
totally modified	1%	2%

Considering the higher enzyme-specific activity as well as the need to maintain consistency with previously reported screening conditions for SARS-CoV-2 PLpro, DTT was employed in the optimized setup.

### Identification of New Inhibitors Using Drug Repurposing Screening Taking into Account PLpro Redox Sensitivity

SARS-CoV-2 PLpro-NAB was screened against three repurposin libraries: Fraunhofer repurposing collection (FhG), EU-OPENSCREEN bioactives collection (EOS), and Dompé “Safe In Man” collection, with 8936 compounds in total.^26^ PR-619 and GRL-0617 were included as reported positive controls for PLpro inhibition.^18,33,34^ All screened plates passed quality control for HTS with a Z’ of >0.5 (mean 0.73) (Figure S2). Compounds were screened at 10 μM, and the percentage of inhibition of the activity of compounds was normalized to the used controls, whereby the effect of PR619 at 20 μM was set to 100% inhibition and DMSO 0.5 v/v % was set to 0% inhibition. 54 compounds with >50% enzyme inhibition were selected for hit confirmation; 50 out of 54 hits were confirmed. False-positive hits causing quenching of the AMC signal were removed from analysis.

As it was already reported that DTT not only stabilizes the protein but also might alter the apparent potency of reactive compounds, we evaluated the inhibitor sensitivity toward different reducing conditions. Here, we pointed out that the effect of DTT may not only result in apparent loss of compound activity on cysteine protease but also in the frequently overlooked capability to react with compounds, leading to the formation of reactive oxygen species and thus nonspecific inhibition of the cysteine protease activity.^35^ Hits were retested in the presence of 1 mM l-cysteine, which has a lower reducing potential. Six compounds retained their inhibitory activity (50% inhibition at 20 μM): CPI-169, semapimod, SRT 1720, sennoside A, purpurogallin, DOM_SIM710 (3′,4’,5′,5,6,7-hexahydroxyflavone), and the positive control PR-619 (Table S1). SRT 1720, which is included in two of the screened libraries, could not be confirmed and was discarded from further analysis. In addition, we confirmed the reference inhibitor GRL-0617 to be active in the l-cysteine buffer.

**Figure 2:**
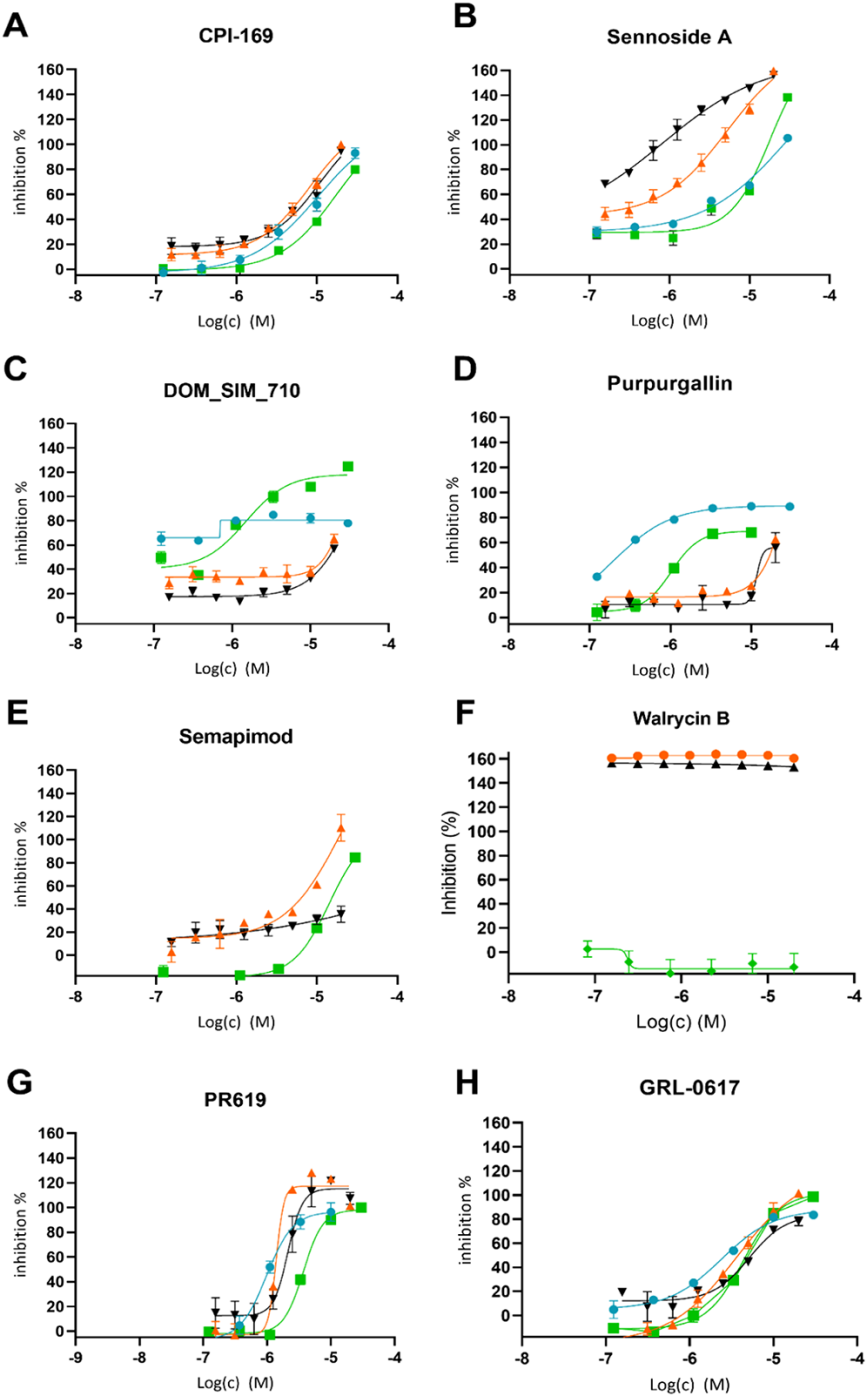
Dose–response analysis of PR-619, GRL-0617, and selected compounds against SARS-CoV-2 PLpro-NAB and PLpro using both DTT and l-cysteine buffers. Values are normalized to controls PR619 at 20 μM (= 100% inhibition) and DMSO (0% inhibition) PLpro-NAB/ISG15 in l-Cys buffer (green); PLpro-NAB/ISG15 in DTT buffer (orange); PLpro/ISG15 in l-Cys buffer (blue); and PLpro/ISG15 in DTT buffer (black) (*n* = 3, data points represent means ± SD). Chemical structures are shown in Figure S4.

Subsequently, the hit compounds were analyzed in dose–response against SARS-CoV-2 PLpro-NAB and PLpro (Figure 2, Table 2). Walrycin B, the most potent hit from the quinone-like series, confirmed a loss of inhibition activity when the reaction buffer contained a mild reductant such as 1 mM l-cysteine, consistent with the previous reports of observed cysteine inactivation by toxoflavin-based compounds through ROS generation.^36^ These findings were corroborated by thermal shift assays (TSA) conducted on PLpro incubated with single doses of quinone-like compounds in the presence or absence of DTT. Without DTT or with l-cysteine, no thermal shift occurred, indicating that compounds do not bind PLpro under these conditions. On the contrary, the combination of DTT and compounds caused a prominent negative shift, indicating a destabilization effect that is explicable with enzyme oxidation (Figure S7). PR-619, CPI-169, semapimod, and GRL-0617 did not show variations in inhibition potency or thermal stability across the different buffers used. In contrast, sennoside A lost its inhibitory potency in the l-cysteine buffer, while purpurogallin and DOM_SIM_710 showed increased inhibition in the same buffer. Furthermore, DOM_SIM_710 and purpurogallin showed higher potency against PLpro compared with PLpro-NAB, whereas semapimod (FhG) displayed activity exclusively against PLpro-NAB, although we could not confirm this result using semapimod from the EOS collection (data not shown).

**Table 2:** Percentage of Inhibition and IC_50_ for PLpro-NAB Was Measured in 1 mM DTT and 1 mM L-Cys Buffers^a^,^b^

Compound name	HC PLpro-NAB inhibition [%] 20 μM triplicates 1 mM DTT buffer	HP PLpro-NAB IC_50_ [μM] triplicates 1 mM DTT buffer	HP PLpro-NAB inhibition [%] 20 μM triplicates 1 mM L-cys buffer	HP PLpro-NAB IC50 [μM] duplicates 1 mM L-cys buffer
sennoside A	159.68 (2.27)	7.01	149.39 (1.26)	18.2
semapimod (EOS)	140.16 (2.99)	1.21	50.46 (6.98)	>20
semapimod (FhG)	118.80 (3.35)	5.23	51.28 (5.64)	14.4
SRT 1720	109.60 (16.57)	>20	169.11 (0.11)	0.82
GRL-0617	105.70 (6.27)	2.12	98.53 (3.03)	4.36
PR-619	101.31 (1.54)	1.39	106.60 (1.05)	3.7
CPI-169 (FhG)	99.81 (2.18)	14.17	70.67 (1.71)	17.47
CPI-169 (EOS)	96.05 (3.31)	19.8	49.41 (2.14)	>20
DOM_SIM_710	65.00 (3.13)	>20	154.95 (1.18)	1,49
purpurogallin	62.76 (1.75)	>20	100.79 (4.49)	1.03

a Values represent averages (±SD)

b Values are normalized to the activity of 20 μM PR619 (positive control) set to 100% inhibition and DMSO set to 0% inhibition. Values of >100% indicate potencies higher than the positive control. HC, hit confirmation; HP, hit profiling.

### Broad-Spectrum Activity and Selectivity of Identified Inhibitors

**Figure 3:**
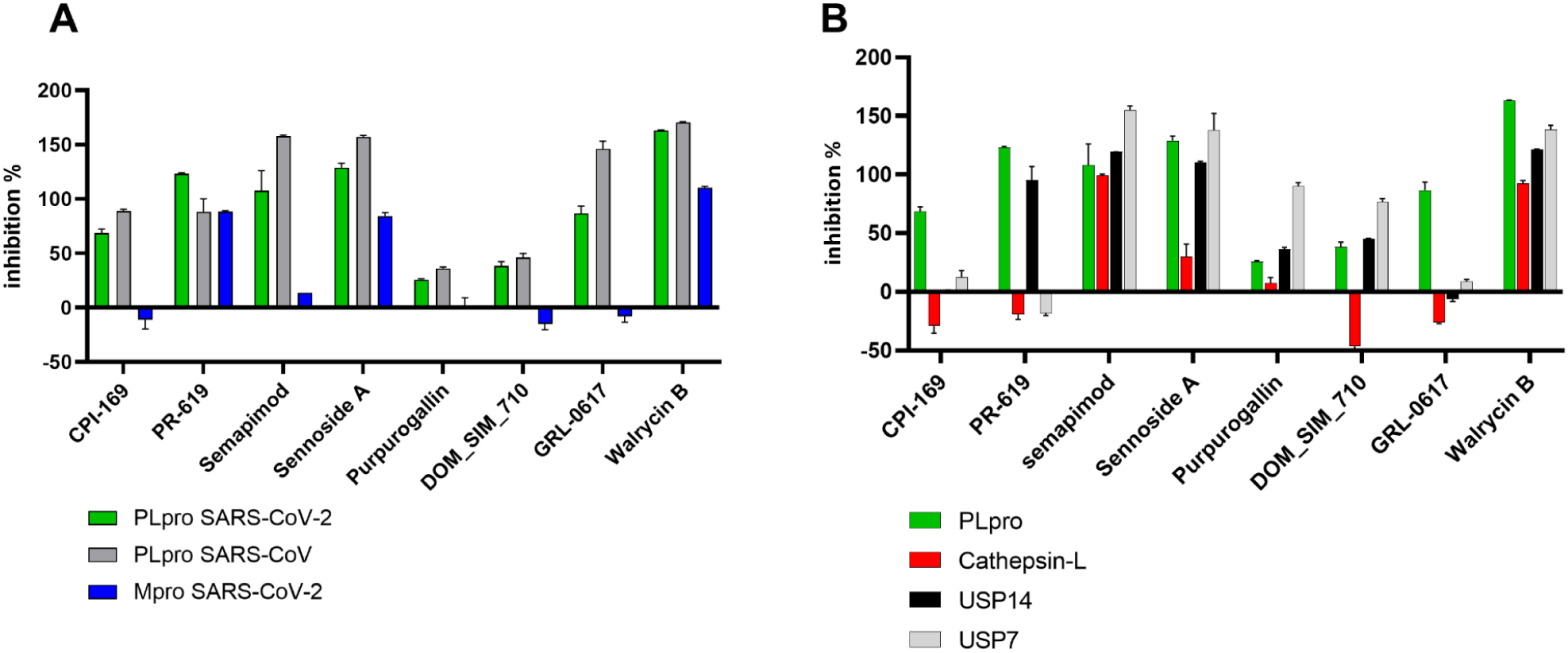
Screening for broad-spectrum inhibition (A) and selectivity (B) of confirmed hits at 10 μM in the presence of 1 mM DTT. Exception: PR-619 was tested at 20 μM against SARS-CoV PLpro (*n* = 3, error bars ±SD).

To assess their specificity or potential broad-spectrum activity, PR-619, CPI-169, semapimod, sennoside A, purpurogallin, and DOM_SIM710 along with GRL-0617 were tested against SARS-CoV PLpro as well as SARS-CoV-2 Mpro. Minimal differences in the inhibitory activity against both SARS-CoV-2 and SARS-CoV PLpro were observed for all compounds. PR-619, sennoside A, and walrycin B showed the inhibition of both proteases from SARS-CoV-2. Notably, the walrycin B inhibition of Mpro was also DTT-dependent, reflecting the same behavior observed with PLpro (Figure S3). The observed broad-spectrum activity of PR-619 and sennoside A aligns with our previously published Mpro repurposing screen (REF),^24^ demonstrating activity under both reducing and nonreducing conditions. CPI-169, semapimod, purpurogallin, DOM-SIM_710, and GRL-0617 exhibited preferential inhibition of PLpro over Mpro (Figure 3A). To explore potential off-target effects on human proteins, we selected the deubiquitinases USP14 and USP7 as the closest homologue of SARS-CoV-2 PLpro and cathepsin-L as representative of the cysteine protease family. With the exception of CPI-169 and GRL-0617, which were selective for the viral proteases, all other compounds inhibited at least one of the human targets, with USP7/USP14 being more affected compared to cathepsin-L (Figure 3B). Walrycin B and semapimod were shown to be active across all of the tested targets.

### Confirmation of CPI-169 Binding to PLpro

**Figure 4:**
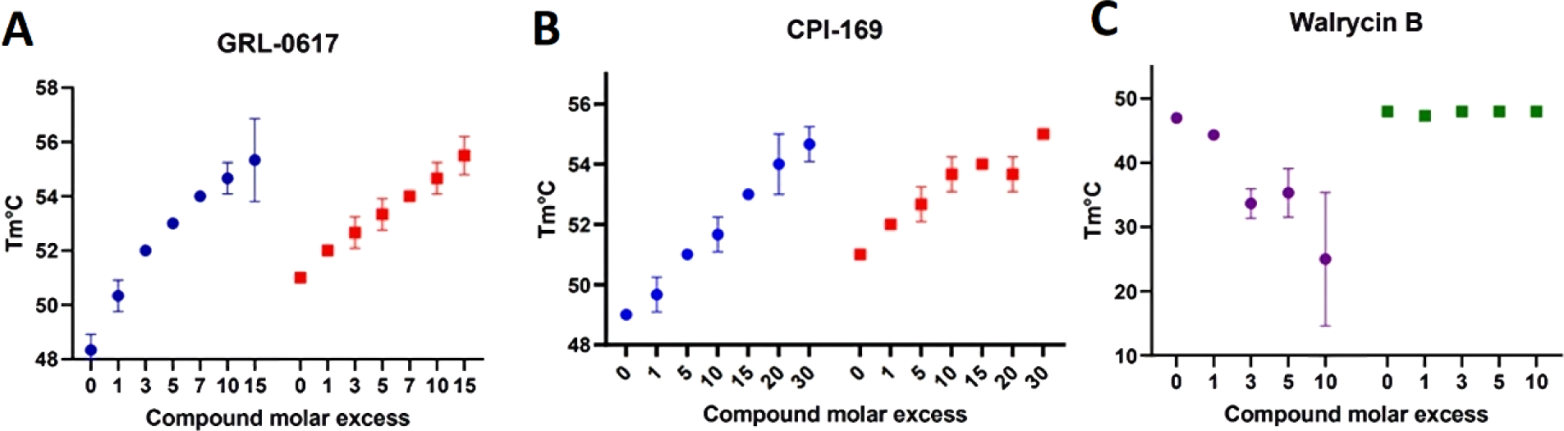
Melting temperature curves of PLpro-NAB (blue) and PLpro-NAB C111S mutant (red) measured in the presence of GRL-0617 (A) and CPI-169 (B). Melting temperature curves of PLpro-NAB (purple) and PLpro-NAB C111S (green) in the presence of Walrycin B and 1 mM DTT (C). *n* = 3, and data points represent mean ± SD.

Hit profiling and selectivity tests confirmed CPI-169 as a promising inhibitor of PLpro. To verify binding to the enzyme, we performed thermal shift assays (TSA) using the PLpro-NAB construct (Figure 4). GRL-0617 was used as a positive control for reported binding to PLpro, while walrycin B served as a reference compound for the oxidative effect and helped in evaluating the inhibition mechanism. Both CPI-619 and GRL-0617 caused a concentration-dependent increase in the Tm value of PLpro-NAB, which was consistent in both DTT and l-Cys buffers. Conversely, walrycin B destabilized the protein in the presence of 1 mM DTT and lost its activity in a mild reducing environment. To verify the putative reactivity of inhibitors toward the active site cysteine, we tested the PLpro-NAB C111S mutant. Both CPI-169 and GRL-0617 increased the Tm of the mutated construct similar to the wild type, whereas the stability of PLpro-NAB C111S remained unaffected in the presence of walrycin B (Figure 4C). Comparable results were obtained using the PLpro and PLpro C111S constructs (data not shown).

We performed a mass mapping characterization to identify if modifications were occurring at the catalytic cysteine in the copresence of compounds and strong reductants. PLpro-NAB was incubated with walrycin B in the presence and absence of DTT. Results were compared with PD119507, a compound known for its redox activity and ability in generating free radicals (Table 3).^35^ In the presence of DTT and the test compounds, the catalytic cysteine appeared highly oxidized as dioxo and trioxo forms. Notably, these effects did not occur when the protein was not exposed to either compound or treated with both compounds in the absence of a reducing agent.

**Table 3:** Percentage of Modification of the Catalytic Cysteine for Walrycin B and PD119507 in the Presence and Absence of DTT Using PLpro-NAB

	Walrycin B		PD119507	
DTT 1 mM	+	-	+	-
No modification	19%	90%	12%	97%
Dioxidation	67%	10%	59%	3%
Trioxidation	13%	0%	30%	0%
Totally modified	81%	10%	88%	3%

### Docking of CPI-169 in the GRL-0617 Binding Pocket

**Figure 5:**
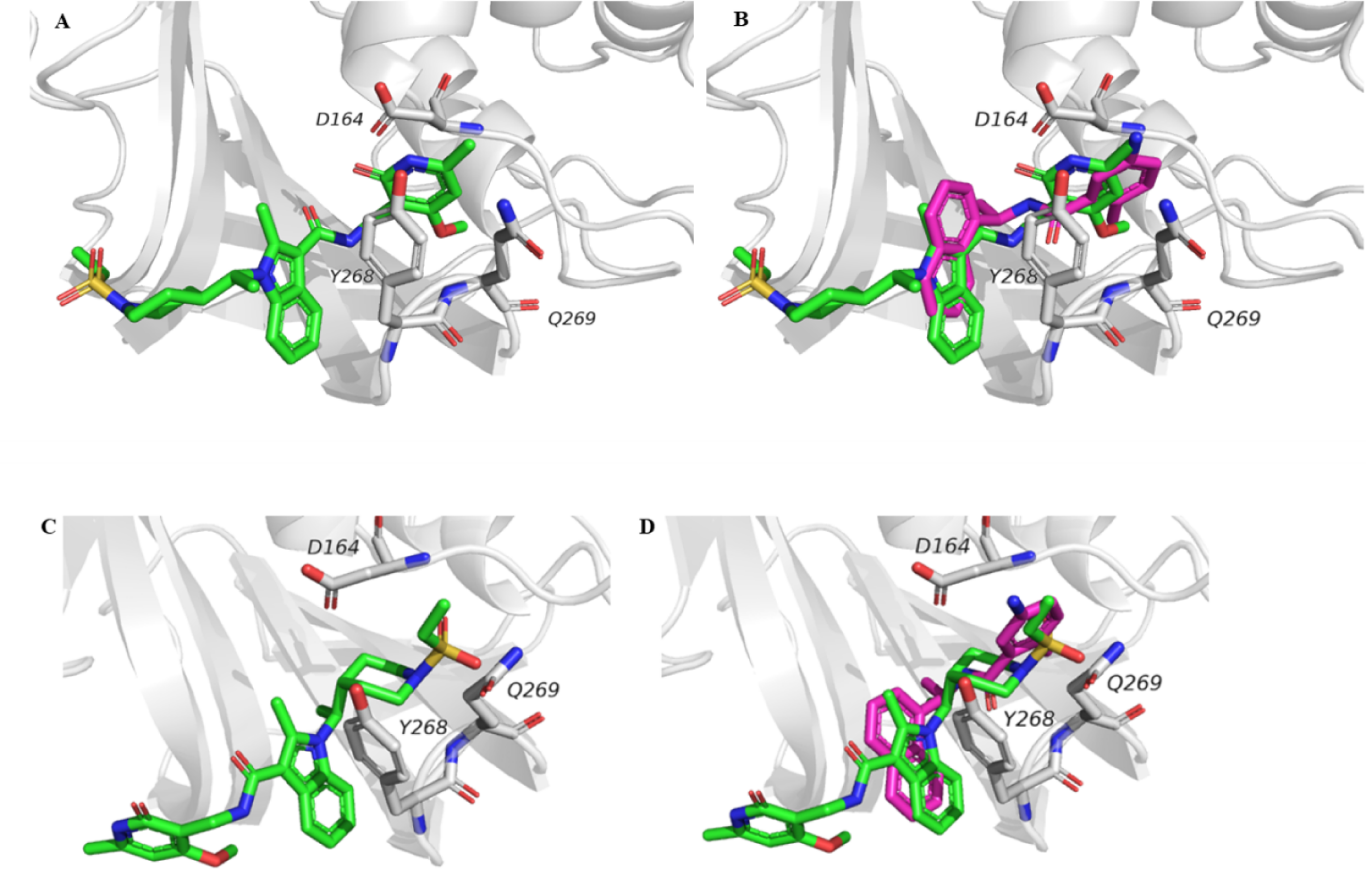
Molecular docking of CPI-169 in the allosteric pocket occupied by GRL-0617. (A) Docking pose 1 of CPI-169 was in the binding pocket of PLpro. (B) CPI-169 pose 1 overlapped with GRL-0617 from the crystal structure (7JRN). (C) Docking pose 2 of CPI-169 in the binding pocket of PLpro. (D) CPI-169 docking pose 2 overlapped with GRL-0617 from the crystal structure (7JRN) and overlapped the CPI-169 docking pose. CPI-169 is colored in green, GRL-0617 in magenta, and PLpro in gray.

Given the biochemical and biophysical similarities between CPI-169 and GRL-0617, we speculated on a similar binding mode. To explore this hypothesis, we conducted docking experiments using PLpro-bound GRL-0617 as a reference crystal structure (PDB:7JRN). We found that CPI-169 can occupy the same allosteric cavity as GRL-0617 (Figure 5). For CPI-169, the two top-ranked docking poses (ChemPLP scoring function) fell within the same range of the reference compound, despite CPI-169 showing a lower potency (for CPI-169, ChemPLP score 1 = 81.9 and ChemPLP score 2 = 90.1; for GRL-0617, ChemPLP score = 86.4). Both CPI-169 poses have similar hydrophobic and polar interactions with the BL2 loop residues Tyr268 and Q269, as observed for GRL-0617 in the 7JRN crystallographic structure. However, they differed in orientation, with pose 1 overlaying the pyridine portion to GRL-0617’s *p*-methyl pyridine ring, while pose 2 has the ethyl sulfone in that site (Figure 5B,D).

### Confirmation of Competitive Binding of CPI-169 in the GRL-0619 Binding Pocket by STD-NMR and Tr-NOESY

**Figure 6:**
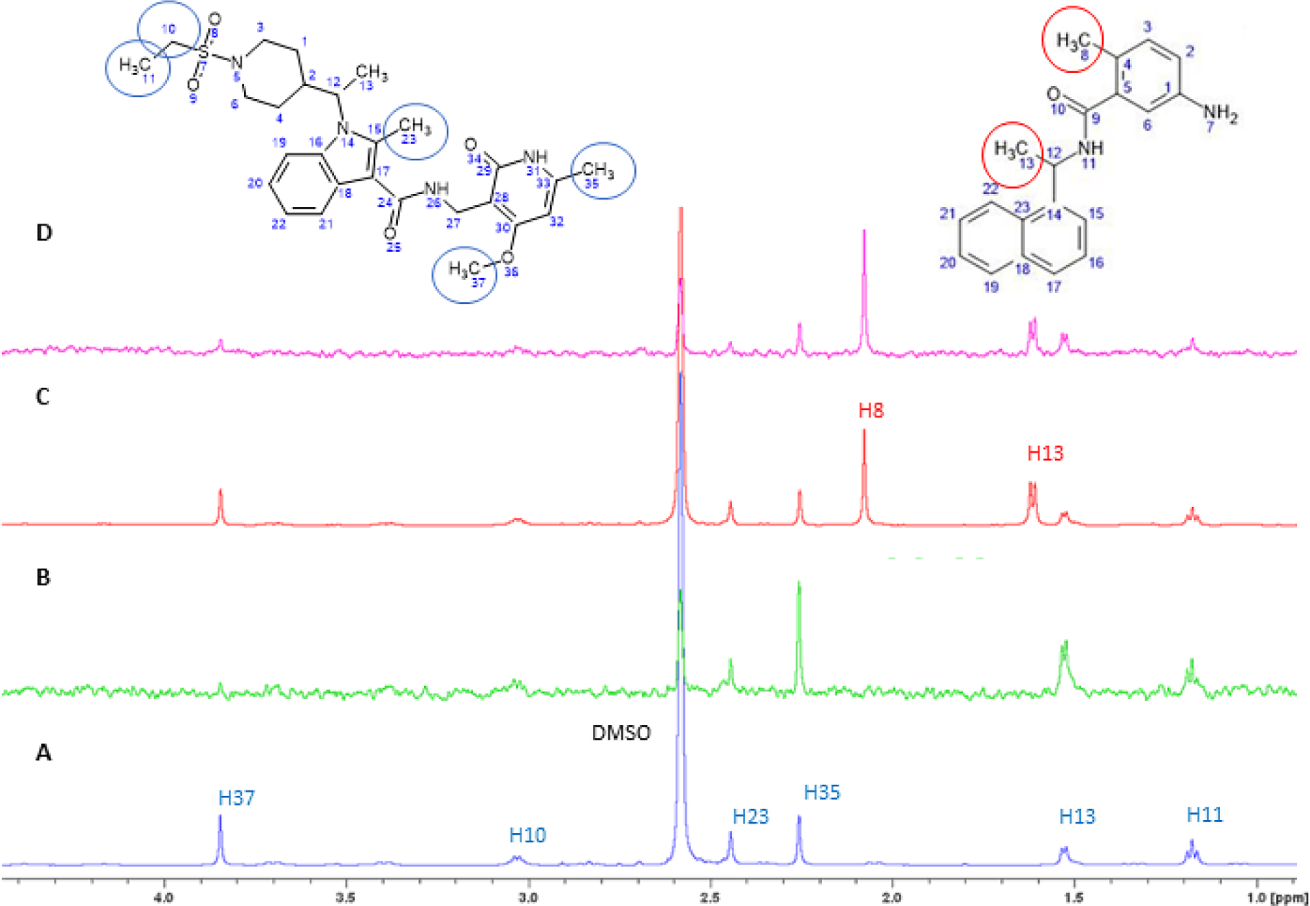
Expanded regions of 1D ^1^H STD spectra showing signals from methyl protons used to monitor the competition between CPI-169 and GRL-0617. The proton signals from CPI-169 (structure on the left) and GRL-0617 (structure on the right) are labeled in blue and red, respectively. (A) 1D ^1^H reference STD spectrum of 0.15 mM CPI-169 recorded at a PLpro-NAB:compound ratio of 1:100. (B) 1D ^1^H STD difference spectrum of 0.15 mM CPI-169 recorded at a PLpro-NAB:CPI-169 ratio of 1:100. (C) 1D ^1^H reference STD spectrum of 0.15 mM CPI-169 recorded at a PLpro-NAB:CPI-169 ratio of 1:100 in the presence of 0.3 mM GRL-0617. (D) 1D ^1^H STD difference spectrum of 0.15 mM CPI-169 recorded at a PLpro-NAB:CPI-169 ratio of 1:100 in the presence of 0.3 mM GRL-0617. The signal of H37 was not taken into account because of its low intensity and absolute error of >10%.

The STD ligand-epitope mapping experiment of CPI-169 confirmed the interaction of the entire molecule with PLpro-NAB, as we observed a rather uniform STD effect over the entire molecule (Figure 5). To confirm the hypothesis of CPI-169 binding in the same GRL-0617 site, a competitive STD-NMR assay was performed (Figure 6). STD amplification factors were calculated for the methyl protons exhibiting STD signals with a sufficient signal-to-noise ratio at a concentration of 0.15 mM of CPI-169 used for the competitive NMR study. The addition of GRL-0167 at a concentration twice that of CPI-169 resulted in a significant reduction in the amplification factors of each methyl proton of CPI-169, indicating the displacement of CPI-169 from the GRL-0617 binding site (Table S3). Our findings suggest a competition of the two ligands for the same binding site. Besides, the intensity of the intramolecular cross-peaks from CPI-169 in the tr-NOESY spectrum matches better with the intramolecular distances predicted by the docking pose 2. Only outliers are cross-signals of H37 with protons of the indole group, suggesting that the pyridine’s methoxy substituent is less proximate to the indole moiety compared to the predicted pose (Table S2). Therefore, the NMR data favor the second predicted pose of CPI-169 over the first one despite the slightly higher PLP score of the first pose. Nevertheless, more detailed structural data are needed for the definitive validation of the binding conformation.

### Effect of CPI-169 on Virus Replication

**Figure 7:**
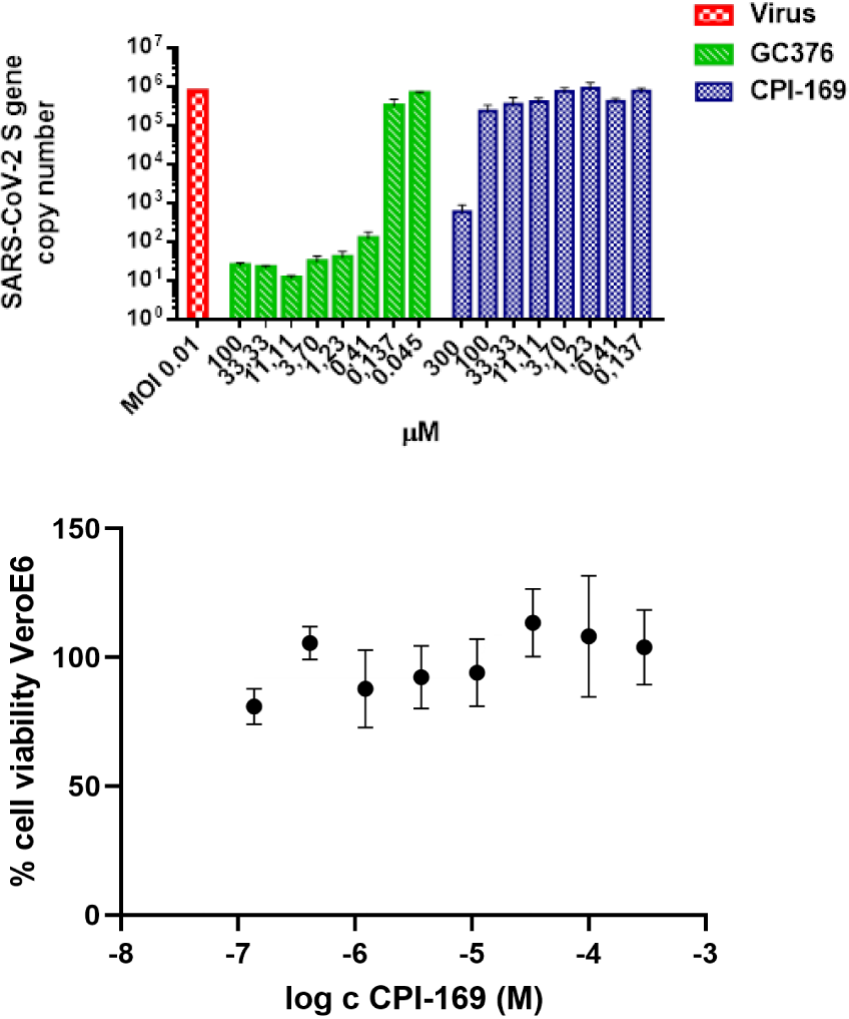
CPI-169 antiviral effect on SARS-CoV-2 replication and cytotoxicity in Vero-E6 cells. Top: Vero-E6 GFP cells were infected with SARS-CoV-2 at an MOI of 0.01 in the presence of indicated concentrations of compounds. Twenty-four h p.i. viral RNA was isolated from the supernatant and processed to quantify the SARS-CoV-2 S protein gene copy number. Bottom: CPI-169 cytotoxic effect on Vero-E6 GFP treated with indicated concentration of compounds, cell viability was measured as the percentage of untreated control. Data points represent the mean ± SEM of three independent experiments.

We evaluated the antiviral effect of CPI-169 in Vero-E6 GFP-expressing cells infected with SARS-CoV-2 (MOI 0.01) using GC376 as a positive reference compound.^37^ At 300 μM, CPI-169 reduced the Spike RNA copy number significantly (Figure 7). To exclude an effect on cell viability, inhibition of cell growth was tested at different compound concentrations after 24 h treatment. No dose-dependent inhibition of cell growth occurred, indicating that the observed S-gene copy number decrease is due to the antiviral effect and not due to cytotoxicity.

## Discussion

SARS-CoV-2 PLpro enzyme plays a crucial role in the viral polyprotein processing and evasion of host immune response, making it a promising target for antiviral therapeutics. Recently, Tan et al. (2024) and Garnsey et al. (2024) identified two GRL-0617-derived antiviral candidates (Jun12682 and PF07957472), clearly demonstrating their efficacy in targeting PLpro in murine models.^19,20^ However, being a Cys-protease with a flexible shallow binding site poses challenges to both rational drug design and the development of robust in vitro screening assays for hit identification.

At the onset of this study, we observed the high sensitivity of PLpro enzyme activity to assay redox conditions, as the addition of reducing agents is mandatory to ensure enzymatic activity and stability. This redox sensitivity aligns with the findings of Arya et al., who demonstrated that DTT (5 mM) prevents aggregation of SARS-CoV-2 PLpro in biochemical assay, although with only minor effect on enzymatic activity.^38^ In our hands, the relatively low specific enzymatic activity of PLpro under nonreducing conditions can be reversed by the addition of reducing agents such as DTT or l-cysteine. Our mass mapping analysis supports the hypothesis that the most likely mechanism behind this phenomenon is the reversible oxidation of cysteines’ SH groups to sulfenic acid, especially of the catalytic Cys111.

To the best of our knowledge, this study represents the first large-scale repurposing screen for SARS-CoV-2 PLpro using its preferred physiologically relevant substrate ISG15 in combination with the target construct containing both the nsp3d (PLpro) and nsp3e (NAB) domains. Primary screening in the presence of DTT resulted in a 0.54% hit rate, in line with the reported low hit rate of PLpro screening campaigns under similar conditions.^17,32^ However, replacing DTT with l-cysteine resulted in a loss of inhibition capacity for a majority of hits. We assume that the inhibition of proteolytic activity by these compounds is due to their reaction with DTT, leading to the nonspecific inhibition of PLpro by oxidation. Previously, Ma et al. (2022) invalidated multiple repurposed compounds, initially reported to inhibit PLpro in biochemical assays.^23^ Here, we confirm the unspecific inhibition of PLpro for many of the compounds selected by the initial DTT-containing screen, providing an explanation related to their reactivity in strong reducing agents such as DTT. Among them, we also identified a group of ortho-quinonoid and para-quinonoid derivatives, such as SF1670 and NSC663284. Previously, this compound class was analyzed for inhibition of CDC25, a subfamily of dual-specificity protein tyrosine phosphatases containing a cysteine in the active site. Due to activity only in the presence of DTT and oxygen, authors assumed a reduction to semiquinone anion radicals, producing reactive oxygen species that result in irreversible cysteine oxidation.^36^ For walrycin B, the most potent inhibitor with DTT-dependent activity, we observed similar reactivity throughout the TSA and mass mapping results.

A small group of compounds, PR-619, CPI-169, semapimod, sennoside A, purpurogallin, and DOM_SIM_710, retained their activity in both l-cysteine and DTT buffer, suggesting that these can specifically inhibit protease activity. By analyzing the broad-spectrum activity toward human target proteins USP14, USP7, and cathepsin-L, as well as viral SARS-CoV-2 Mpro and SARS-CoV PLpro, we revealed selective inhibition of PLpro only for CPI-169 that also proved to reduce viral replication in cells with no evident cytotoxicity. Using TSA and ligand-based NMR, we showed a similar behavior of CPI-169 compared to GRL-0617, previously proposed by Shen et al.^39^ Both compounds bound to the wild type as well as the C111S mutant of PLpro, suggesting a catalytic cysteine111-independent binding and indirectly supporting a noncovalent nature of the CPI-169 inhibition. This hypothesis was proved by STD-NMR assay, by which we were able to show that CPI-169 binds to the same site as GRL-0617. NMR results match well with the binding mode obtained by molecular docking (Figure 5C), highlighting weaker interactions with PLpro with respect to GRL-0617, supporting the lower potency observed for CPI-169 (Figure S6). At present, the GRL-0617 compound stands as the primary scaffold for lead optimization, yielding notable noncovalent and covalent derivatives, such as compound XR8–24 and the Jun-series described in the recent publication by Tan et al., enhancing the inhibitory activity through a structural-based drug design approach, as demonstrated in in vitro and in vivo models.^19,40^ Given the shared binding pocket with GRL-0617, we consider CPI-169 as an additional scaffold for lead development. Studies using GRL-0617 have already demonstrated the efficacy of targeting the BL2 groove site to inhibit PLpro, with potential for extending interactions toward the catalytic core and proximal Ub binding site (Val70, Leu71). Although in the current state, it is less potent compared to newly designed inhibitors, we believe CPI-169 optimization can provide a novel tool for PLpro inhibitors design.

## Supporting Information Available

Kinetic PLpro and PLpro-NAB parameters (Figure S1), QC of the primary screening (Figure S2), walrycin B inhibition ± DTT (Figure S3), and chemical structures of the active compounds (Figure S4); hit compounds tested in different conditions (Table S1); report NMR results (Table S2 and Figures S5 and S6); 2D interaction scheme with PLpro active site (Figure S6); TSA results of selected compound on both PLpro and PLpro-NAB ± DTT (Figure S7) (PDF)
