# Supplementary material for: Thiol-Reactive or Redox-Active: Revising a Repurposing Screen Led to a New Invalidation Pipeline and Identified a True Noncovalent Inhibitor Against Papain-like Protease from SARS-CoV-2
Source: ACS Pharmacol Transl Sci. 2024 Oct 4;8(1):66–77. doi: 10.1021/acsptsci.4c00325 (PMC11729419; doi:10.1021/acsptsci.4c00325)
Supplement: Supplementary file 1 — pt4c00325_si_001.pdf [file pt4c00325_si_001.pdf]

Supplementary material

Thiol-reactive or redox-active: Revising a repurposing screen led to a new invalidation-pipeline and identified a true non-covalent inhibitor against papain-like protease from SARS-CoV-2.

Maria Kuzikov[a,j,*,#], Stefano Morasso[b,k,#], Jeanette Reinshagen[a], Markus Wolf[a], Vittoria Monaco[c,d], Flora Cozzolino[c,d], Simona Golič Grdadolnik[e], Primož Šket[f], Janez Plavec[f], Daniela Iaconis[g], Vincenzo Summa[h], Angela Corona[i], Annalaura Paulis[i], Francesca Esposito[i], Enzo Tramontano[i], Maria Monti[c,d], Andrea R. Beccari[g], Candida Manelfi[g], Björn Windshügel[a,j], Philip Gribbon[a], Paola Storici[b,*] and Andrea Zaliani[a]

[a]*Fraunhofer Institute for Translational Medicine and Pharmacology ITMP, Discovery Research ScreeningPort, Schnackenburgallee 114, 22525 Hamburg, Germany.*
[b]*Protein Targets for Drug Discovery Lab, Elettra-Sincrotrone Trieste S.C.p.A., SS 14 - km 163,5 in AREA Science Park 34149 Basovizza, Trieste, Italy.*
[c]*Department of Chemical Sciences, University of Naples "Federico II" Comunale Cinthia, 26, 80126 Naples, Italy.*
[d]*CEINGE Advanced-Biotechnologies "Franco Salvatore", Via Gaetano Salvatore, 486, 80145 Naples, Italy.*
[e]*Laboratory for Molecular Structural Dynamics, National Institute of Chemistry, Hajdrihova 19, 1000 Ljubljana, Slovenia.*
[f]*Slovenian NMR Center, National Institute of Chemistry, Hajdrihova 19, 1000 Ljubljana, Slovenia.*
[g]*EXSCALATE - Dompé Farmaceutici SpA, via Tommaso De Amicis 95, 80131, Napoli, Italy.*
[h]*Department of Pharmacy, University of Naples Federico II, Via D. Montesano, 49, 80131 Naples, Italy.*
[i]*Dipartimento di Scienze della vita e dell'ambiente, Cittadella Universitaria di Monserrato, SS-554, Monserrato, 09042 Cagliari, Italy.*
[j]*Constructor University, School of Science, Campus Ring 1, 28759 Bremen, Germany.*
[k]*Department of Chemical and Pharmaceutical Sciences, University of Trieste, Via Licio Giorgeri 1, 34127 Trieste, Italy*

[*]Corresponding authors:
Maria Kuzikov, maria.kuzikov@itmp.fraunhofer.de
Paola Storici, paola.storici@elettra.eu

# M.K and S.M. contributed equally to this work

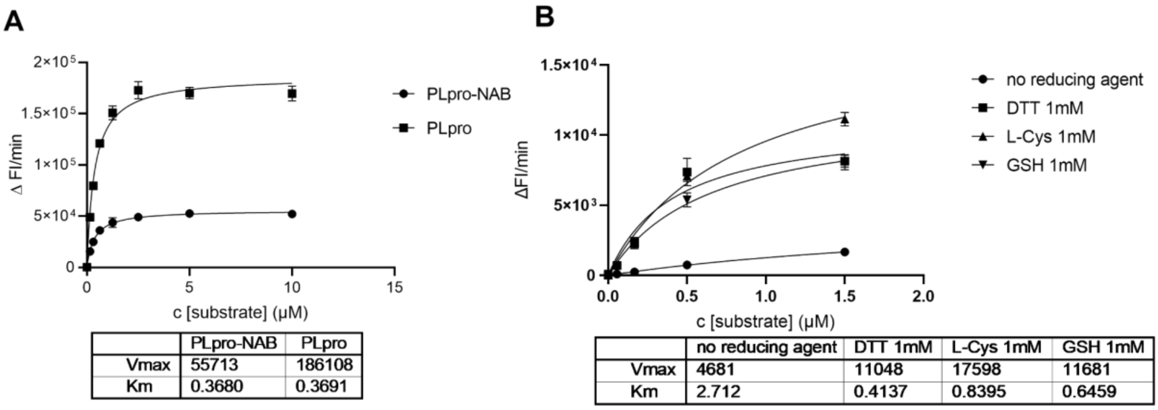

	PLpro-NAB	PLpro
Vmax	55713	186108
Km	0.3680	0.3691

	no reducing agent	DTT 1mM	L-Cys 1mM	GSH 1mM
Vmax	4681	11048	17598	11681
Km	2.712	0.4137	0.8395	0.6459

Figure S1. Key kinetic parameters of SARS-CoV-2 PLpro. A: Km and Vmax of PLpro containing the NAB domain (PLpro-NAB) and PLpro catalytic domain (PLpro). B: Kinetic parameters of PLpro-NAB in an assay buffer containing different reducing agents. (N= 3, error bars +/-SD)

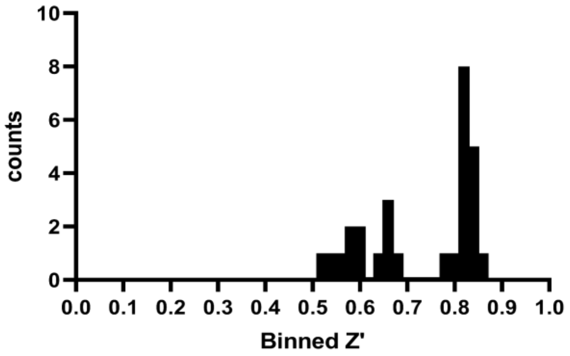

FigureS2. Quality control of SARS-CoV-2 PLpro-NAB primary screening. All calculated Z' values > 0.5 showing robust assay, calculated S/B ratio value of ~ 2.1, whereby S refers to the DMSO control and B refers to values observed using 20 μM PR-619 as inhibitor.

Table S1. Nominal hit compounds from PLpro-NAB primary screen with > 50 % inhibition, tested for activity under different reducing conditions. Compounds active in DTT and L-cysteine buffer are marked in red, controls are marked in bold. Values represent averages (± SD).

Compound Name	HC PLpro-NAB SARS-CoV-2 inhibition [%] 20 µM triplicates DTT BUFFER	HP PLpro-NAB SARS-CoV-2 IC50 [µM] triplicates DTT BUFFER	HP PLpro-NAB SARS-CoV-2 inhibition [%] 20 µM triplicates L-CYS BUFFER	HP PLpro-NAB SARS-CoV-2 IC50 [µM] duplicates L-CYS BUFFER	Interference inhibition [%] 20 µM based on product interference DTT BUFFER
Acriflavinium Hydrochloride	27.57 (6.10)	12.44	-99.55 (4.20)	>20	-17.48
CPI-169 (FhG)	99.81 (2.18)	14.17	70.67 (1.71)	17.47	-0.73
Sodium-Tanshinone-ii-A-Sulfonate	124.17 (1.49)	4.67	-51.93 (2.1)	>20	-7.60
YM-155	138.51 (0.85)	17.86	-5.61 (6.66)	>20	-0.69
BVT-948	154.88 (0.27)	0.39	-16.22 (16.3)	>20	-137.72
BYK-204165	137.58 (2.88)	6.17	7.10 (11.9)	>20	-5.54
Chicago-Sky-Blue-6b	51.02 (1.78)	>20	3.64 (5.37)	>20	-5.59
**PR-619**	**101.31 (1.54)**	**1.39**	**106.60 (1.05)**	**3.70**	**-14.28**
Delavirdine (mesylate)	52.21 (2.63)	>20	-69.33 (9.08)	>20	-20.91
Homidium Bromide	149.01 (1.72)	13.69	18.32 (8.81)	>20	1.40
Alpha Lapachone	146.26 (3.11)	4.43	-6.97 (8.44)	>20	-0.29
Semapimod (FhG)	118.80 (3.35)	5.23	51.28 (5.64)	14.40	-5.72
Pyrithione Zinc	80.42 (4.73)	20.41	16.01 (7.04)	>20	3.55
Propidium-Iodide	135.55 (3.03)	0.24	14.90 (7.41)	>20	0.27
TAS-103 (dihydrochloride)	154.91 (1.47)	5.94	17.75 (9.12)	>20	1.87
Ryuvidine	159.90 (1.27)	0.26	-33.69 (4.70)	>20	-2.15
NSC-663284 (FhG)	150.90 (0.34)	0.22	7.56 (11.27)	>20	-0.99
Walrycin B (FhG)	160.54 (0.89)	0.04	7.90 (6.75)	>20	4.84
Ro-08-2750	119.29 (1.83)	0.40	-33.99 (8.82)	>20	0.17
Cyanocobalamin	141.27 (3.32)	2.90	32.29 (7.95)	>20	4.81
Sennoside A	159.68 (2.27)	7.01	149.39 (1.26)	18.20	1.69
Bacitracin (Zinc)	67.28 (8.42)	10.25	6.99 (8.69)	>20	-1.67
6-Hydroxy-DL-DOPA	81.47 (1.21)	>20	4.81 (4.61)	>20	14.95
Rose Bengal Sodium	106.67 (2.42)	>20	-32.41 (2.42)	>20	12.07
Purpurogallin	62.76 (1.75)	>20	100.79 (4.49)	1.03	12.84
ML113	74.45 (0.96)	>20	-21.50 (0.87)	>20	10.70
Juglone	83.67 (1.88)	>20	-14.79 (1.62)	>20	12.84
PD081125	186.10 (4.22)	0.58	1.14 (2.64)	>20	16.52
PD119507	186.40 (4.35)	0.065	-16.42 (5.8)	>20	3.43
ML120	82.78 (2.58)	3.14	-56.07 (10.54)	>20	3.32
PT 1	87.07 (2.59)	9.32	-36.09 (5.10)	>20	9.93

Compound Name	HC PLpro-NAB SARS-CoV-2 inhibition [%] 20 µM triplicates DTT BUFFER	HP PLpro-NAB SARS-CoV-2 IC50 [µM] triplicates DTT BUFFER	HP PLpro-NAB SARS-CoV-2 inhibition [%] 20 µM triplicates L-CYS BUFFER	HP PLpro-NAB SARS-CoV-2 IC50 [µM] duplicates L-CYS BUFFER	Interference inhibition [%] 20 µM based on product interference DTT BUFFER
9,10-Phenanthrenequinone	180.94 (3.91)	0.504	-40.79 (2.42)	>20	-2.46
PD086277	180.40 (3.94)	0.807	-30.11 (4.13)	>20	4.30
PD011561	49.68 (1.79)	>20	-34.83 (6.92)	>20	13.27
Semapimod (EOS)	140.16 (2.99)	1.21	50.46 (6.98)	>20	9.91
SRT 1720	109.60 (16.57)	>20	169.11 (0.11)	0.82	22.23
Beta-Lapachone	175.40 (2.95)	4.71	-37.39 (5.44)	>20	13.40
Cryptotanshinone	40.70 (8.42)	>20	-71.36 (3.20)	>20	3.37
Dihydrotanshinone I	80.74 (1.17)	0.92	-176.06 (5.05)	>20	-55.01
Menadione	115.37 (7.44)	11.5	-45.68 (4.33)	>20	-9.41
Etoposide	37.84 (2.75)	>20	-45.18 (11.25)	>20	16.29
Evans Blue	126.18 (2.52)	8.68	-67.82 (3.17)	>20	6.47
SF1670	183.62 (4.27)	0.25	-32.73 (4.18)	>20	9.17
NSC 663284 (EOS)	168.59 (3.49)	2.15	-38.39 (8.32)	>20	14.95
Hexachlorophene	109.33 (1.27)	>20	-18.33 (5.18)	>20	15.14
3-Methyl Toxoflavin	181.38 (6.76)	0.074	-26.71 (3.86)	>20	10.44
Resazurin (sodium salt)	72.88 (4.56)	>20	-36.51 (7.18)	>20	11.91
Walrycin B (EOS)	181.14 (4.17)	0.069	-35.90 (1.44)	>20	8.01
ML240	93.76 (13.43)	>20	-11.93 (4.14)	>20	16.72
CPI-169 (EOS)	96.05 (3.31)	19.8	49.41 (2.14)	>20	9.34
DOM_SIM_710	65.00 (3.13)	>20	154.95 (1.18)	1.49	10.43
**GRL-0617**	**105.70 (6.27)**	**2.12**	**98.53 (3.03)**	**4.36**	**2.12**

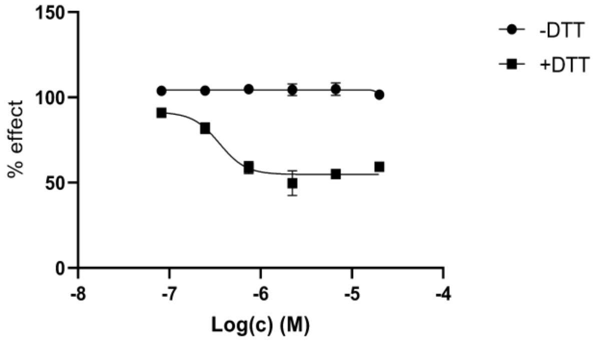

Figure **S3.** Dose dependent inhibition of SARS-CoV-2 Mpro using Walrycin B in presence and absence of DTT.

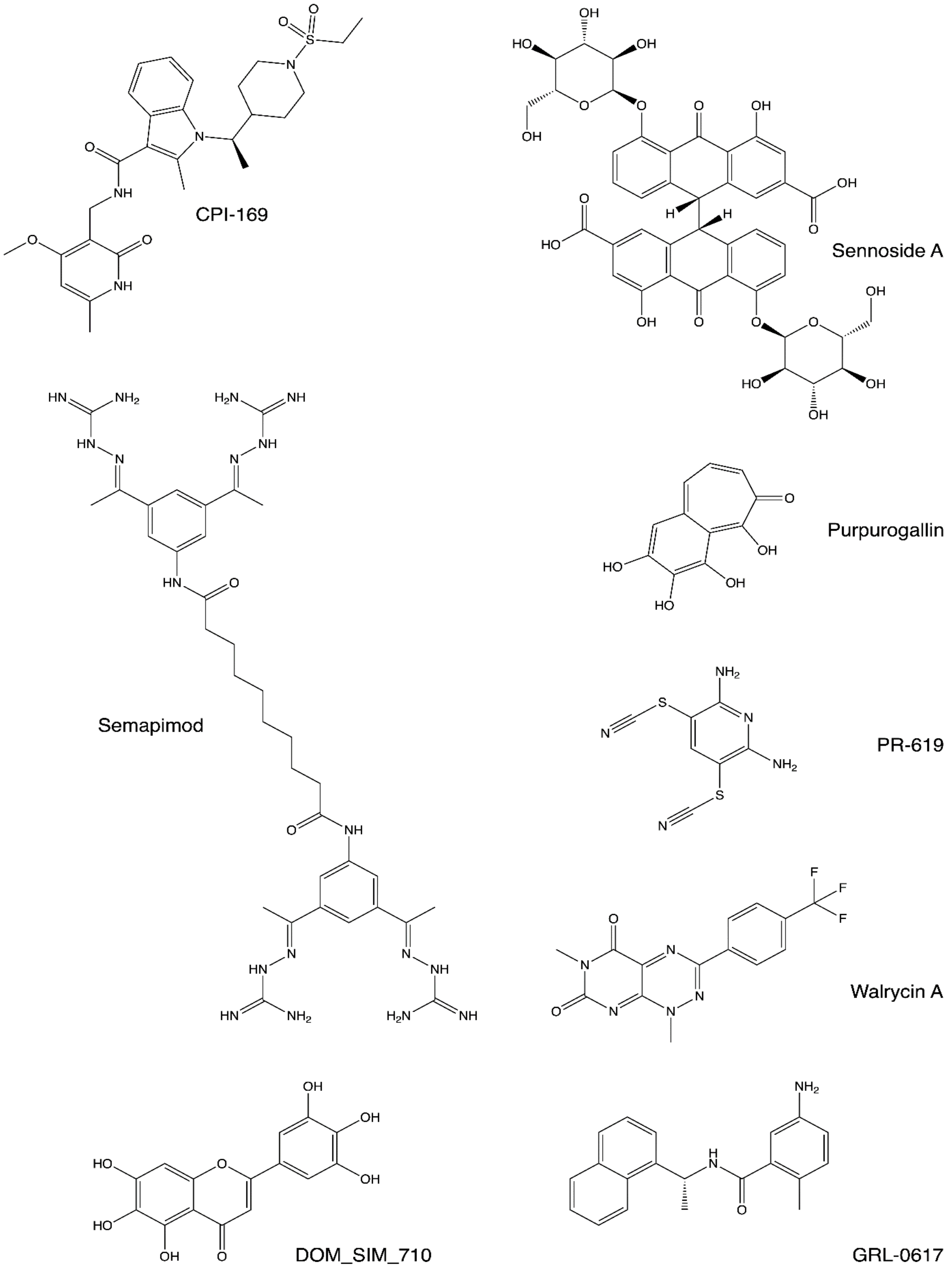

Figure S4. Chemical structures of PR-619, GRL-0617 and compounds that retained the inhibition activity against SARS-CoV-2 PLpro-NAB and PLpro under both DTT and L-Cysteine buffers.

Table S2. The non-trivial NOEs of CPI-169 at a ligand-PLpro ratio of 1:100 observed in tr-NOESY spectrum and distances from the model structure of CPI-169 in complex with PLpro. Very weak NOEs of H37 observed only at reduced threshold indicate that the methoxy group has a different orientation than in the model, away from H26, H21, H22.

**NOE cross-peak**	**Intensity of NOE cross-peak**	**Distance from the model structure of CPI-169 in complex with PLpro-NAB (Å)**
H13-H19	strong	3.3 (C13-H19)
H2-H19	strong	2.3
H23-NH26	weak	4.8 (C23-NH26)
H37-H32	strong	3.0 (C37-H32)
H12-H19	weak	5.0
H27-NH26	medium	2.6, 2.9
H27-H21	weak	3.6, 4.3
H21-H37	very weak	3.1 (H21-C37)
H22-H37	very weak	4.2 (H22-C37)
H26-H37	very weak	2.3 (H26-C37)
H3/6-H11	weak	4.0, 5.2 (C11-H3/6)
H23-H13	strong	3.9 (C23-C13)
H23-H1/4	medium	2.9, 3.7 (C23-H1/4)

Table S3. STD amplification factors of 0.15 mM CPI-619 at a PLpro/compound concentration of 1:100 (AMP), and STD amplification factors of CPI-169 in the same protein/compound ratio in presence of 0.3 mM of GRL-0617 (AMP'), with relative absolute errors. AMPs of the methyl groups of CPI-169 decrease in presence of the GRL-0617, demonstrating they compete for the binding.

Proton	AMP	AMP Error (%)	AMP' with GRL-0167	AMP' Error (%)	AMP'/AMP
H11	0.74	4	0.44	3	0.59
H13	1.19	2	0.81	1	0.68
H23	0.43	4	0.27	4	0.63
H35	0.89	1	0.55	2	0.62

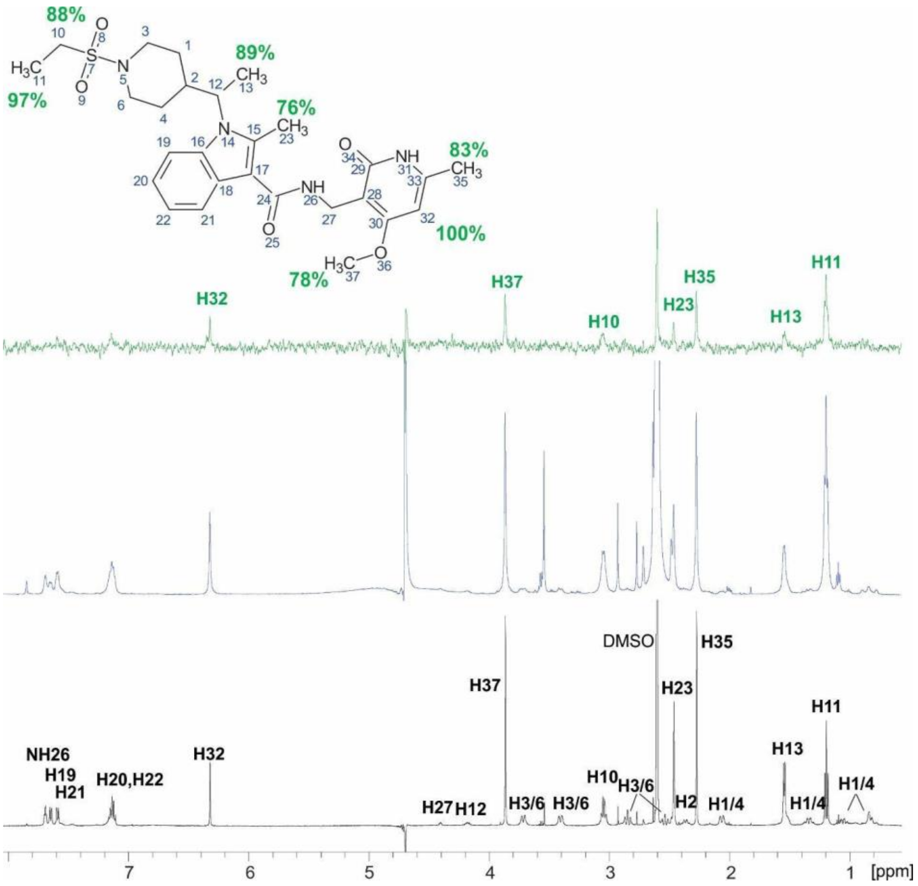

Figure S5. (In black) 1D ^{1}H spectrum of CPI-169 showing assignment of proton chemical shifts. (In green) 1D ^{1}H difference STD spectrum of CPI-169 at a PLPro-NAB:ligand ratio of 1:100. The STD amplification factors were calculated for the signals with sufficient signal-to-noise ratio marked in green and normalized to the intensity of the signal with the largest STD effect. Above, the molecular structure illustrates the proton nomenclature and the relative degrees of saturation of the individual protons. (In blue) 1D ^{1}H reference (off resonance) STD spectrum.

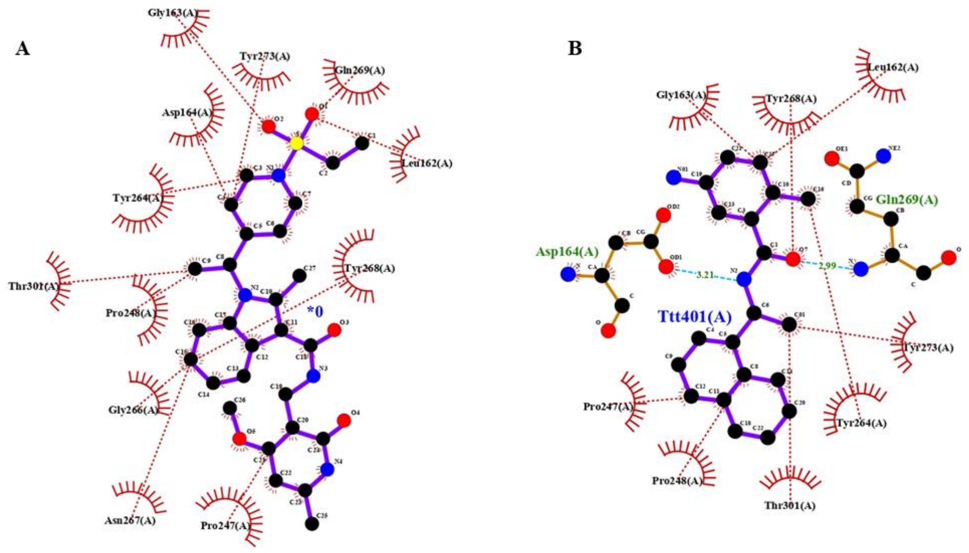

Figure S6. 2D scheme of interactions between CPI-169 (A) and GRL-0167 (B) with PLpro

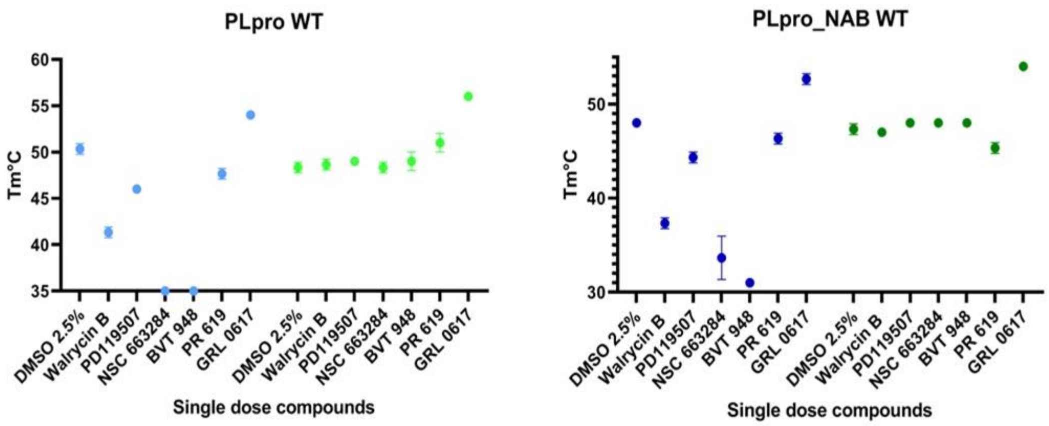

Figure S7 Melting temperature curves of PLpro (left) and PLpro-NAB (right) measured in presence of 10x single-dose of quinone-like compounds and positive controls PR-619 and GRL-0617, in presence of 1 mM DTT (light blue and dark blue respectively) or absence (light green and dark green respectively). (N= 3, error bars +/-SD)
